# Isolation of Mycosporine-like Amino Acids from Red Macroalgae and a Marine Lichen by High-Performance Countercurrent Chromatography: A Strategy to Obtain Biological UV-Filters

**DOI:** 10.3390/md21060357

**Published:** 2023-06-10

**Authors:** Julia Vega, Daniela Bárcenas-Pérez, David Fuentes-Ríos, Juan Manuel López-Romero, Pavel Hrouzek, Félix López Figueroa, José Cheel

**Affiliations:** 1 Centro Experimental Grice Hutchinson, Lomas de San Julián, Instituto Andaluz de Biotecnología y Desarrollo Azul (IBYDA), Universidad de Málaga, 2, 29004 Málaga, Spain; felixlfigueroa@uma.es; 2 Laboratory of Algal Biotechnology—Centre ALGATECH, Institute of Microbiology of the Czech Academy of Sciences, Opatovický mlýn, 379 81 Třeboň, Czech Republic; barcenas@alga.cz (D.B.-P.); hrouzek@alga.cz (P.H.); 3 Faculty of Science, University of South Bohemia, Branišovská 1760, 370 05 České Budějovice, Czech Republic; 4 Department of Organic Chemistry, Faculty of Sciences, University of Malaga, Campus de Teatinos s/n, 29071 Málaga, Spain; davidfuentesrios31@gmail.com (D.F.-R.); jmromero@uma.es (J.M.L.-R.)

**Keywords:** countercurrent chromatography, isolation, marine lichen, mycosporine-like amino acids, photoprotection, red macroalgae

## Abstract

Marine organisms have gained considerable biotechnological interest in recent years due to their wide variety of bioactive compounds with potential applications. Mycosporine-like amino acids (MAAs) are UV-absorbing secondary metabolites with antioxidant and photoprotective capacity, mainly found in organisms living under stress conditions (e.g., cyanobacteria, red algae, or lichens). In this work, five MAAs were isolated from two red macroalgae (*Pyropia columbina* and *Gelidium corneum*) and one marine lichen (*Lichina pygmaea*) by high-performance countercurrent chromatography (HPCCC). The selected biphasic solvent system consisted of ethanol, acetonitrile, saturated ammonium sulphate solution, and water (1:1:0.5:1; *v*:*v*:*v*:*v*). The HPCCC process for *P. columbina* and *G. corneum* consisted of eight separation cycles (1 g and 200 mg of extract per cycle, respectively), whereas three cycles were performed for of *L. pygmaea* (1.2 g extract per cycle). The separation process resulted in fractions enriched with palythine (2.3 mg), asterina-330 (3.3 mg), shinorine (14.8 mg), porphyra-334 (203.5 mg) and mycosporine-serinol (46.6 mg), which were subsequently desalted by using precipitation with methanol and permeation on a Sephadex G-10 column. Target molecules were identified by HPLC, MS, and NMR.

## 1. Introduction

Solar radiation reaching the Earth’s surface consists of about 5% ultraviolet (UV) radiation, of which 95% is UV-A (λ = 315–400), 5% is UV-B (λ = 280–315 nm), 40% is visible radiation (λ = 400–700 nm), and the rest is infrared radiation [1]. The negative effects of UV radiation on human health are well-known, although this type of radiation also has beneficial properties for health, such as promoting vitamin D synthesis or regulating serotonin levels, among others [2,3,4,5]. UV-B rays are the most harmful type of radiation that reach the Earth´s surface. They can be absorbed by DNA, forming thymine dimers, which can cause mutations associated with skin cancer. UV-A radiation has indirect adverse effects on DNA through the formation of reactive oxygen species, which have been associated with premature photoaging [6,7,8]. In general, the effects caused by UV radiation can be divided into short-term (e.g., sunburn, erythema, or photoallergies) and long-term (e.g., keratosis, skin cancer, immunosuppression, or photoaging) [7,9]. Photoprotection is necessary to reduce the risks of UV radiation effects. There are several strategies for this, such as wearing appropriate clothing, hats, or glasses, reducing sun exposure during the central hours of the day, and the use of sunscreens.

Sunscreens consist of various UV filters, that protect against both UV-B and UV-A. These filters are usually divided into physical or inorganic that reflect the UV radiation (e.g., titanium dioxide or zinc oxide) and chemical, organic or synthetic that absorb UV radiation and dissipate it as heat (e.g., octocrylene, octilmetoxicinamate, ethylexcyl triazone or bisoctrizole) [10]. In recent decades, several studies have shown that certain UV filters can accumulate in the aquatic environment and can have negative effects on the ecosystem (e.g., reducing phytoplankton growth, triggering coral bleaching, disrupting hormone balance in fish or mammals) [11,12,13,14]. It has also been found that some chemical filters can have negative effects on human health, such as photoallergies, phototoxicity, or disruption of the endocrine system [15,16], and some of them may also lack efficacy because the absorption of UV radiation can cause the cleavage of chemical bonds, leading to the formation of photodegradation products and molecular instability [17].

Mycosporine-like amino acids (MAAs) are secondary metabolites found in organisms that are highly exposed to sunlight in clear waters or intertidal systems (e.g., cyanobacteria, red macroalgae, marine lichens, or corals) and are mainly involved in protection against UV radiation. MAAs are a diverse group of low molecular weight nitrogen-containing compounds (<400 Da) that are colourless, hydrophilic, and with the ability to absorb UV-B and UV-A radiation. There are more than 30 types of MAAs, all of which share the same central structure, a cychlohexenone (with an oxygen double bond in C1) or cyclohexenimine (with a nitrogen double bond in C1) chromophore, which is responsible for UV absorption. The different types of MAAs differ in their nitrogen substituents in the chromophore, which determines their specific absorption spectra, which varies between 309 and 360 nm. Depending on the chromophore and substituent, MAAs can be divided into two groups: (1) oxo-MAAs, which have a single nitrogen substituent conjugated to the C3 of the cyclohexenone ring, e.g., mycosporine-glycine, mycosporine-taurine, or mycosporine-serinol, which absorb in the UV-B region, and (2) imino-MAAs having two nitrogen substituents in the C1 and C3 of the cyclohexenimine ring, e.g., palythine, asterina-330, palythinol or porphyra-334, which absorb in the UV-A region. Another group of derivatized MAAs has been recently described, including sulphated or glycosidated MAAs [18,19,20,21,22,23]. These molecules are potential candidates for use in cosmetic products as natural or biological UV filters because, in addition to their capacity to absorbed UV radiation, they have other beneficial properties for the skin (e.g., antioxidant, anti-inflammatory, or antiaging), are nontoxic and biodegradable, and present high photo- and thermostability [20,21,23,24,25,26,27,28,29,30,31,32]; however, few products based on MAAs are currently available on the market. Most of them are based on extracts from *Porphyra umbilicalis* (Rhodophyta) with MAAs and antioxidant properties, such as Helioguard^TM^365 or Helionori^®^.

In recent decades, different authors have tried to purify MAAs using different techniques. Some have used simple chromatography columns, such as silica gel or ion exchange columns [26,27], although these techniques usually reduce the extraction yield and require multiple and tedious steps. Other authors worked with semipreparative HPLC columns [33], although the cost of the equipment and columns may limit their use. Countercurrent chromatography (CCC) is an attractive separation technique that uses two immiscible liquid phases (as stationary and mobile phases) and no solid support [34]. In recent years, numerous articles and reviews have been published on the separation of natural products, such as flavonoids, polyphenols, pigments, or fatty acids [35,36,37,38,39,40,41], using this technique. In the absence of a solid support, CCC offers several advantages over solid-phase chromatography (e.g., HPLC), such as no irreversible adsorption, high loading capacity, high recovery of the injected sample, low risk of sample denaturation, and low solvent consumption [42]. CCC is generally used for the separation of apolar or moderately polar compounds, whereas the isolation of polar compounds (such as proteins, polysaccharides, or MAAs) has been less studied. In the case of MAAs, few authors have attempted to use this technique for their isolation/fractionation [43,44,45].

The aim of this work was to isolate five different MAAs from aqueous extracts of two macroalgae and one marine lichen by high-performance countercurrent chromatography (HPCCC) and to demonstrate the use of a multiple sequential injection method to increase the throughput of the process.

## 2. Results and Discussion

**Figure 1:**
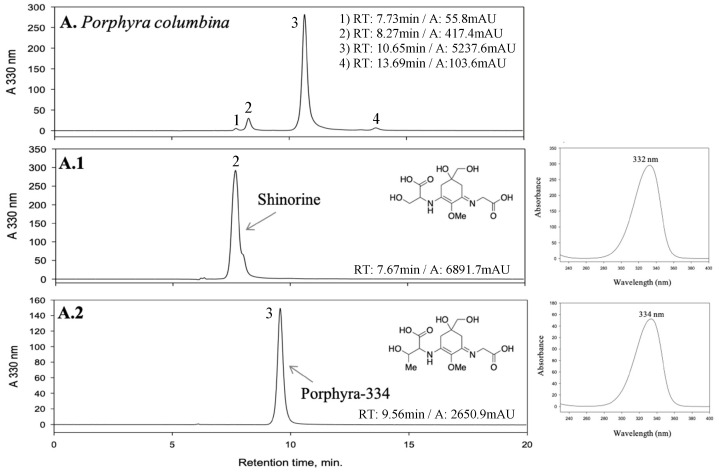

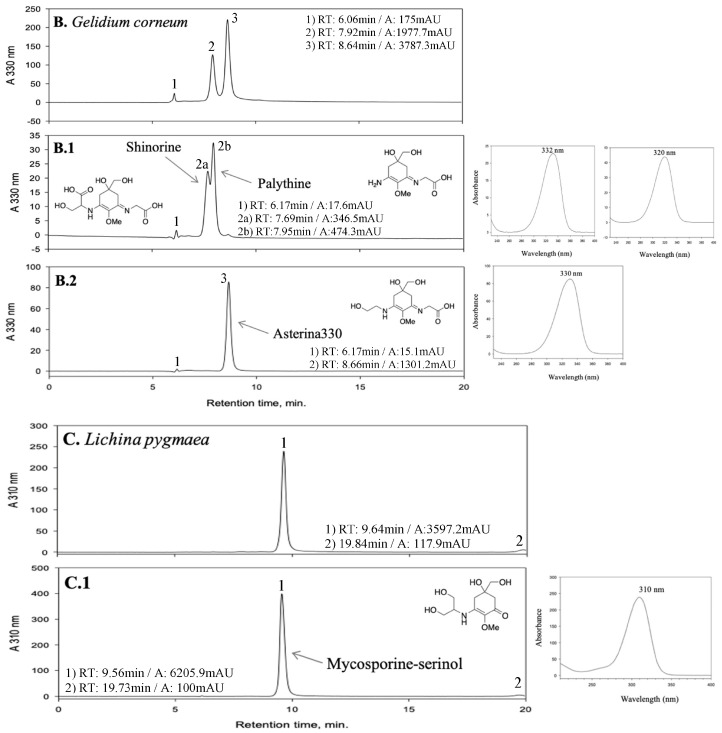
HPLC chromatograms of two red macroalgae (*Pyropia columbina* and *Gelidium corneum*) and a marine lichen (*Lichina pygmaea*) extracts, and the fractions obtained enriched in mycosporine-like amino acids (MAAs) from each extract, with the retention time (RT) and area (**A**) of the peaks. The UV-spectrum, maximum absorbance and chemical structure of each identified MAA is also present. (**A**) *Pyropia columbina* extract, (**A.1**) Shinorine-enriched fraction, (**A.2**) Porphyra-334-enriched fraction; (**B**) *Gelidium corneum* extract, (**B.1**) Palythine-enriched fraction (with shinorine), (**B.2**) Asterina-330-enriched fraction; (**C**) *Lichina pygmaea* extract and (**C.1**) Mycosporine-serinol-enriched fraction.

The yield of the different aqueous extractions was about 20% for all species. Figure 1A–C show the chromatograms of the individual extracts. The extract of *Pyropia columbina* (Figure 1A) showed two major peaks in the chromatogram, identified as shinorine (SH; λ_max_: 332 nm) and porphyra-334 (P-334; λ_max_: 334 nm). The predominant MAA was P-334 (>90%). The *Gelidium corneum* extract (Figure 1B) has two peaks identified as palythine (Pal; λ_max_: 320 nm) and asterina-330 (Ast-330; λ_max_: 330 nm). In this case, the content of Ast-330 was almost 2.5-fold higher than that of Pal (70 versus 30%). Finally, *Lichina pygmaea* (Figure 1C) had only one peak, identified as mycosporine-serinol (M-ser; λ_max_: 310 nm). *P. columbina* had the highest content of MAAs (31.25 ± 2.93 mg g^−1^ dry extract (DE) = 6.25 mg g^−1^ dry biomass (DB)), followed by *L. pygmaea* (15.40 ± 0.83 mg g^−1^DE = 3.08 mg g^−1^DB) and *G. corneum* (6.32 ± 0.27 mg g^−1^DE = 1.26 mg g^−1^DB). Similar results were observed by other authors in species of the same genus. In *Pyropia columbina (=Porphyra columbina)*, *Phycocalidia acanthophora (=Pyropia acanthophora), Porphyra umbilicalis* and *Neopyropia elongata (=Pyropia elongata)*, different authors determined values ranging from 5.2 to 10.6 mg g^−1^DB [46,47,48,49]. All observed that the predominant MAAs were P-334 and SH. In *Gelidium* sp., the same authors observed values around 0.3–6.8 mg g^−1^DB and greater variability in MAAs patterns. In *L. pygmaea* [27,50], values of 1.1 mg g^−1^DB of a MAA that absorbed at 310 nm were observed. Initially, this MAA was identified as mycosporine-glycine by HPLC [27], although more recent works have identified it as M-ser [31] by MS. Roullier et al. [43], who also identified M-ser in *L. pygmaea*.

The selection of the biphasic solvent system is the most important part of the HPCCC procedure. The separation of highly polar compounds, such as MAAs, requires the development of aqueous biphasic solvent systems. Such systems can be prepared by adding inorganic salts to increase the density differences between the two phases. In this study, two different salts were tested: ammonium sulphate and sodium chloride. The addition of a saturated sodium chloride solution did not lead to the formation of two phases, while the addition of ammonium sulphate showed good results (Table 1). A suitable biphasic solvent system must provide a good partition coefficient (0.5 ≤ *K* ≤ 3.5) to allow the isolation of the target compounds [51,52]. The system used must also ensure equilibrium, i.e., sufficient retention of the stationary phase inside the column, as long as the settling time is lower than 30 s and the density difference between the two phases higher than 0.08 g mL^−1^ [51,52]. Table 1 shows the physical and chemical properties of the different biphasic solvent systems tested. Systems 1, 4 and 5 meet these requirements. In a first attempt, system 5 was selected due to the lower toxicity of acetone in comparison with acetonitrile; however, when this system was transferred to the equipment, the stationary phase was not retained sufficiently inside the column. System 4 (without acetonitrile) was also tested but showed the same limitation as system 5. Finally, system 1 was selected.

Different parameters were optimized for the HPCCC separation method. The flow rate was set at 3 mL min^−1^ because, at a higher flow rate, the pressure increased too much and the equipment started to lose liquid phases due to overpressure (Table 2). The rotational speed of the column was set to the maximum speed of the equipment (1600 rpm). The *a priori* retention time of the target compounds was calculated for the different species. In *P. columbina*, compounds **1** and **2** should elute at 37.1 and 43.2 min, respectively. In *G. corneum*, peak 1 should elute at 40.0 min and peak 2 at 42.3 min. In *L. pygmaea*, the compound should elute at 52.1 min. This information is useful to know (*a priori*) if the compounds can be separated properly, an estimation of the amount of solvent required for the chromatographic process, and also provides information on the duration of the HPCCC run. Only in the case of *G. corneum*, both compounds would elute very close, will it not allow a large increase in the sample loading.

**Figure 2:**
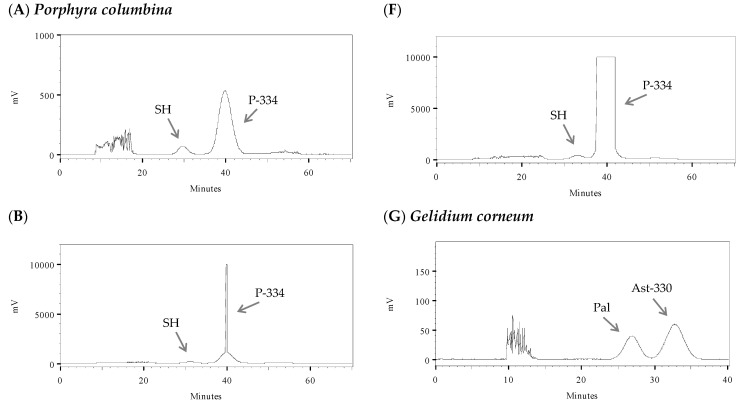

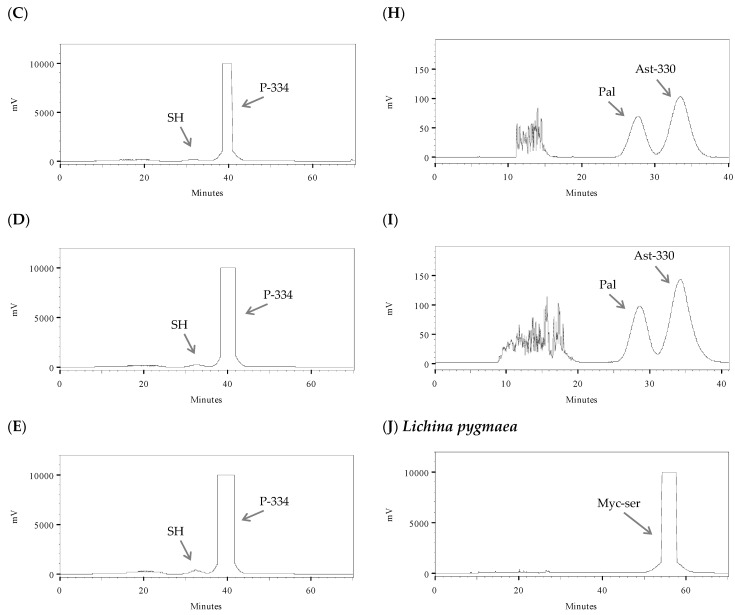
High-performance countercurrent chromatography (HPCCC) optimization using different sample loadings and loop capacities for the isolation of mycosporine-like amino acids (MAAs). (**A**–**F**) *Pyropia columbina* with shinorine (SH) and porphyra-334 (P-334) peaks: (**A**) 200 mg of sample loading in 3 mL loop, (**B**) 400 mg of sample loading in 3 mL loop, (**C**) 600 mg of sample loading in 3 mL loop, (**D**) 800 mg of sample loading in 6 mL loop, (**E**) 1000 mg of sample loading in 6 mL loop, and (**F**) 1200 mg of sample loading in 6 mL loop. (**G**–**I**): *Gelidium corneum* with palythine (Pal) and asterina-330 (A-330) peaks: (**G**) 100 mg of sample loading in 2 mL loop, (**H**) 200 mg of sample loading in 2 mL loop, and (**I**) 300 mg of sample loading in 2 mL loop. (**J**) *Lichina pygmaea* with mycosporine-serinol peak (M-ser), 1200 mg of sample loading in 6 mL loop. Column temperature: 30 °C; rotational speed: 1600 rpm; detection: 330 nm in *P. columbina* and *G. corneum* and 310 nm in *L. pygmaea*.

Different approaches were taken to optimize sample loading, depending on the species. In the case of *P. columbina* (Figure 2A–F; Table 3 (A–F)), sample loading optimization started from 200 to 600 mg in 3 mL. At this point, the resolution of the peaks was good, but the solution reached its saturation point, so a higher volume of the loop was tested to increase the sample loading. Sample loadings of 800 to 1200 mg were tested with a 6 mL loop. Good separation of target compounds was observed at 800 and 1000 mg, while lower resolution was obtained at 1200 mg; therefore, the optimal injection was selected at 1000 mg in 6 mL of LP. In the case of *G. corneum*, sample loading optimization started at 100 to 300 mg because the target peaks eluted closely (Figure 2G–I; Table 3 (G–I)). A decrease in peak resolution was observed when 300 mg was injected, so the optimal injection was selected at 200 mg in 2 mL of LP. In the case of *L. pygmaea* (Figure 2J; Table 3 (J)), which only showed one peak, the highest amount of dry extract (1200 mg DE) to be dissolved in 6 mL LP was injected. In Figure 1, the HPLC analysis of the fractions obtained by HPCCC processes showed good separation of most target compounds. Palythine eluted closely with shinorine only in the case of *G. corneum*.

**Figure 3:**
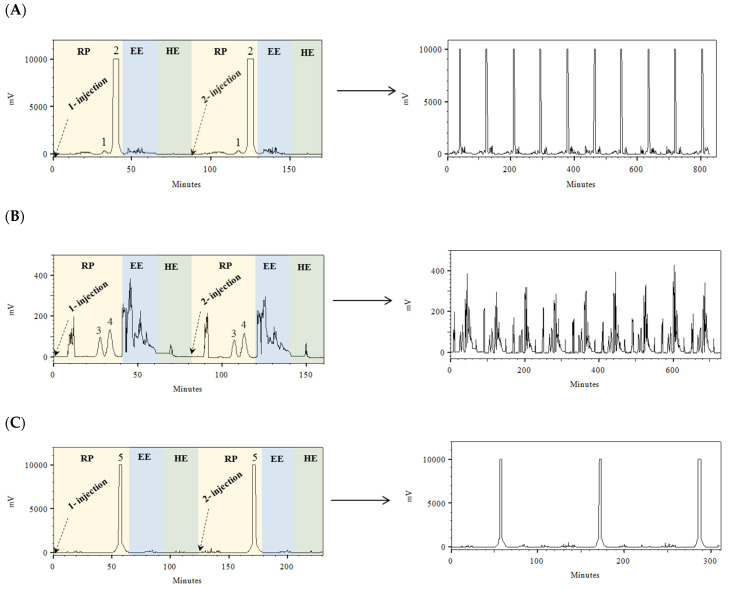
Development of two sequential injections and multiple injections in high-performance countercurrent chromatography (HPCCC) to obtain different mycosporine-like amino acids from two red macroalgae ((**A**): *Pyropia columbina* and (**B**): *Gelidium corneum*) and a marine lichen ((**C**): *Lichina pygmaea*): (1) shinorine; (2) porphyra-334; (3) palythine; (4) asterina-330 and (5) myc-serinol. (**A**) *P. columbina*, (**B**) *G. corneum* and (**C**) *L. pygmaea*.

In order to increase the throughput of the process, a continuous isolation method using multiple sequential injections with an elution–extrusion mode (EE) was studied [40]. The development of two sequential injections for each species is shown in Figure 3. After separation of the target compounds from the extracts of *P. columbina*, *G. corneum*, and *L. pygmaea*, the EE was applied for 20 min. EE allows for the complete replacement of the stationary phase inside the column by a new one. After that, the hydrodynamic equilibrium was achieved (20 min), and a new separation cycle could be performed. These results indicated that more sample injections could be performed for the isolation of the different MAAs; therefore, following this methodology, a multiple sequential injection HPCCC method was applied (Figure 3). In the case of *P. columbina* and *G. corneum*, eight sequential injections were performed; however, in the case of *L. pygmaea*, only three sequential injections were possible because higher sample loading was achieved, and little collected biomass was available. The total sequential multiple injections lasted 675, 635, and 325 min for *P. columbina*, *G. corneum*, and *L. pygmaea*, respectively. In the three cases, the time included 15 min for the first filling of the column and 20 min for the first hydrodynamic equilibrium. If the same number of injections were performed with *L. pygmaea* as in the other species, the time would be 875 min. The developed method consumed 2.13 L of solvents in the case of *P. columbina*, 2.01 L of solvents in the case of *G. corneum*, and 1.08 L of solvents in the case of *L. pygmaea* (if the extract of *L. pygmaea* were injected the same times as in the other species, 2.73 L of solvents would be consumed). The developed HPCCC method afforded five different MAA fractions enriched with Pal (1.94 mg), Ast-330 (2.76 mg), SH (14.76 mg), P-334 (203.46 mg) and M-ser (46.63 mg) with recoveries of 63.9, 50.7, 50.1, 92.3 and 84.1%, respectively. The chromatograms of the different MAA-enriched fractions are observed in Figure 1. The relative standard deviation (RSD) between the obtained MAAs in the different separation cycles was 6.7, 5.8, 5.5, 13.6 and 3.3% for Pal, Ast-330, SH, P-334 and M-ser, respectively. In most cases, good reproducibility was obtained, and only P-334 showed an RSD greater than 10, indicating greater variability between separation cycles [40].

As the biphasic solvent system contained a saturated solution of ammonium sulphate, the obtained MAA-enriched fractions had a high content of this salt. To remove it, two steps were performed: (1) precipitation with methanol and (2) size-exclusion chromatography with Sephadex G-10. The cleanings with methanol resulted in the removal of a large portion of the salt, although a loss of the target compounds were observed. To remove the remaining salt, size exclusion chromatography was used with Sephadex G-10, which has the smallest particle size (40–120 μm) and molecular exclusion limit (>700 g mol^−1^) on the market. After chromatography, two different fractions were obtained from each fraction enriched with MAAs (Figure S1, Supplementary material). In the desalted MAA fractions, the amount of salt was reduced by about 90%, and the recoveries after this process were 22, 57, 28, 35, and 27% for Pal, Ast-330, SH, P-334, and M-ser, respectively (Figure S2). For size-exclusion chromatography, separation was based on molecular weight (Pal: 244.24 g mol^−1^; Ast-330: 288.30 g mol^−1^; SH: 332.31 g mol^−1^; P-334: 346.33 g mol^−1^; M-ser: 262.13 g mol^−1^; ammonium sulfate: 132.14 g mol^−1^), e.g., the MAA Pal, which has the lowest molecular weight, similar to that of ammonium sulphate, showed the lowest recovery with 22%. Other variables, such as the amount of salt in the sample of the structure of the molecule, could influence the permeation in the Sephadex G-10. The desalting process resulted in a large loss of target molecules, so further studies are needed to find a suitable desalting process. The use of other techniques, such as dialysis, was not possible due to the low molecular weight of MAAs and ammonium sulphate. Nanofiltration could be tested in the future, although a disadvantage of this technique is the cost and maintenance of the membranes [53].

Identification of target molecules was performed by mass spectrometry (MS) and nuclear magnetic resonance (NMR) in addition to HPLC. MS confirmed the separation of target compounds, but in some cases a combination of MAAs was detected (Figure S3). Initially, only SH and P-334 were detected in *P. columbina* extracts by HPLC; however, after the HPCCC procedure, MS also identified Pal in the SH-enriched fraction. In the HPLC chromatogram of the SH-enriched fraction, a small shoulder can also be seen, which can be associated with Pal. Something similar happened in the Pal-enriched fraction of *G. corneum*. Initially, HPLC identified only Pal and SH in the *G. corneum* extract. Nevertheless, after separation by HPCCC, HPLC detected a double peak in the Pal-enriched fractions with a λ_max_ at 332 nm corresponding to SH. MS also identified SH in the Pal-enriched fraction. Thus, the methodology used in this work (HPLC and HPCCC) was not suitable for the separation of Pal and SH. As described in the bibliography, column, eluent, solvent, or methodology can affect the separation of peaks [54,55,56].

These results were also confirmed by NMR [57] (Figure S4). The ^1^H NMR spectrum of each fraction showed the singlets corresponding to the methoxy group (3.5–3.8 ppm), the methylene of the primary alcohol group (2.3–2.5 ppm), and the methylene adjacent to the imine nitrogen atom (3.6–3.9 ppm) present in the common MAA structure, except in the case of the oxo-MAAs, which have no imine moiety. Only in the case of the fraction enriched with Ast-330, the NMR analyses did not provide clear information. In addition to the usual signals, the ^1^H NMR spectrum of the P-334-enriched fraction showed the characteristic multiplet attributed to the proton present in the side chain of P-334 (at about 4.0 ppm), confirming the presence of this MAA in this fraction. For the fraction enriched with SH, the ^1^H NMR spectrum showed the characteristic AB system (4.3–4.7 ppm) attributed to the HOCH_2_CHCO structure in the side chain of SH, confirming the presence of this MAA as the main component of this fraction. In addition, some minor signals were observed in this spectrum, which can be attributed to the presence of Pal as a minor component. The ^13^C NMR spectrum of this fraction showed characteristic signals at 177.5, 177.0, and 167.8 ppm corresponding to both carbonyl and imine groups, which also confirmed the presence of SH. The ^1^H NMR spectrum of the Pal-enriched fraction confirmed the presence of Pal as the major component and SH to likely be a minor compound. In this fraction, the characteristic doublet signals at 2.88 and 3.32 ppm, attributed to the CH_2_OH group of Pal, along with a broad singlet at 7.14 ppm, attributed to the free amino group of this MAA, are clearly evident. The presence of two signals at 99.8 and 105.7 ppm in the ^13^C NMR spectrum of this fraction, characteristic of the enamine group, confirmed the presence of Pal in this fraction. Similarly, in the ^1^H NMR spectrum of the M-ser-enriched fraction, the CH_2_OH system was observed at 3.11 and 3.46 ppm, occurring with a larger chemical shift than expected, probably due to the anisotropic effect of the free carbonyl group in a chromophore of the mycosporine structure. In addition, two multiplet signals were observed at about 3.7 and 3.5 ppm, which can be attributed to the presence of a serinol group in the mycosporine skeleton. Finally, the presence of two signals at 175.1 and 177.4 ppm in the ^13^C NMR spectrum assigned to the C=O and COOH groups of the molecule confirms the presence of a M-ser derivative as the main compound in this fraction. Based on HPLC chromatograms and MS identification, the purity of the MAAs obtained from *P. columbina* were higher than 90%, SH presented a purity of 92% with small traces of Pal, and P-334 showed a purity of 98%. In *G. corneum* a mix of two MAAs was obtained: the Pal-enriched fractions presented with 40% of SH and 56% of Pal (in total, 96% of MAAs), whereas Ast-330 showed a purity of 97%. In *L. pygmaea* a value 98% of M-ser was observed; however, according to the ^1^H NMR integrals, it can be said that the obtained MAA-enriched-fractions presented a purity higher than 80% (except in case of Ast-330, in which no good RMN results were obtained).

Some authors have reported the isolation of MAAs using liquid–liquid chromatography. Roullier et al. [43] used centrifugal partition chromatography (CPC) for the purification of M-ser from *L. pygmaea*. The biphasic solvent system BuOH-AcOH-H_2_O (4:1:5; *v*:*v*:*v*) was used in a multiple dual-mode inside a CPC to obtain a fraction containing the compound, which was subsequently isolated by HPLC using a C18 column. Roullier et al. reported a good purity of M-ser but did not provide data on recovery. Orfanoudaki et al. [44] also used CPC (Heptane-EtOAc-BuOH-MeOH-H_2_O), but only to fractionate a methanolic extract of *Agarophytum chilensis* before HPLC purification of the MAAs, so they did not provide any information on the recovery rate or purity of the MAAs obtained. Recently, Zwerger et al. [45] worked with an aqueous biphasic system for the purification of MAAs by CPC. They used a biphasic solvent system (H_2_O-EtOH-(NH_4_)_2_SO_3_-MeOH (51.4:28:18.2:2.4; *w*:*w*:*w*:*w*) similar to the one used in this work, although they used it in an opposite mode (LP-stationary phase and UP-mobile phase). They achieved separation of P-334 and SH from *Porphyra* sp. in 90 min, followed by solid-phase extraction (SPE), and achieved 85–90% of recovery. By using the ascending mode, they were able to reduce the amount of salt in the fractions, although the time required increased. In our case, we separated SH and P-334 from *P. columbina* faster (45 min) than Zwerger et al. and achieved similar recoveries of SH and P-334 (80–85%); however, in contrast to these authors, we did not achieve good recovery rates after the desalting process. It is likely that the high salinity in our samples made the separation between ammonium sulphate and MAAs difficult and resulted in a large loss of our target molecules. Compared to the abovementioned studies and despite the difficulties in separating MAAs from ammonium sulphate, we achieved a faster separation process and were able to obtain five different MAAs, including oxo-MAAs with λ_max_ in UV-B (M-ser) and imino-MAAs with λ_max_ in UV-A (Pal, Ast-330, SH and P-334). The isolation of two of the mentioned MAAs (Pal and Ast-330) have not yet been reported in the bibliography. In the separation process developed, multiple-sequential injections were performed in the HPCCC, which would increase the amount of biomass processed per hour or day; however, further research is needed to develop a suitable purification method using CCC for the isolation of MAAs. Removing ammonium sulphate (without increasing the time required for separation) or developing new biphasic solvent systems without salts is still a challenge, as is achieving good separation between Pal and SH or separating other MAAs, such as palythinol.

An efficient and easily scalable purification of various MAAs from different sources is necessary to drive the development of new biological UV filters based on these molecules. CCC is a scalable and cost-effective technology [58]. The combination of MAAs with different absorption maxima and a natural broadband UV filter can protect the UV-B and UV-A regions. De la Coba et al. [31] previously reported a cosmetically stable topical sunscreen based on purified MAAs by adsorption and ion exchange chromatography [27]. The formulation, which contained 4% P-334 (+SH) and 3% M-ser, achieved a SPF of 8.4, similar results to the reference cream, which contained synthetic UV-filters in similar proportions (2.6% octylmethoxy-cinamate and 2.0% Butilmetho-xybenzoylmethane). In vivo studies on mouse skin have shown that MAAs can prevent UV-induced skin damage (sunburn cells, malpighian or dermal and hypodermal thickening), by inhibiting the expression of p53 and caspase-3 (protein indicators of apoptosis). Thus, topical application of MAAs contributed to the maintenance of the skin’s antioxidant defence system and Hsp70 expression (potential biomarker of acute UV damage) [59,60,61,62]. In addition to UV protective properties, MAAs have other beneficial properties for the skin, such as antioxidant [20], inhibition of matrix metalloproteinases (MMPs) and collagenases [28,32,63,64], wound-healing properties [29,32], immunomodulatory effects [30,65,66], reduction of DNA damage [25,67] and proliferation of fibroblasts [68].

## 3. Materials and Methods

### 3.1. Chemicals and Reagents

Methanol (HiPerSolv Chromanorm, VWR Inc., Fontenay-sous-Bois, France) and acetic acid (AnalaR Normapur, VWR Inc., Fontenay-sous-Bois, France) were used for the HPLC analysis. Absolute ethanol (AnalaR Normapur, VWR Inc., Fontenay-sous-Bois, France), acetonitrile (HiPerSolv Chromanorm, VWR Inc., Fontenay-sous-Bois, France), acetone (HiPerSolv Chromanorm, VWR Inc., Fontenay-sous-Bois, France) and ammonium sulfate ((NH4)2SO4) (Lach-ner s.r.o., Neratovice, Czech Republic) were used for the performance of HPCCC.

### 3.2. Biological Material

Three different species were used in this study. The red macroalgae *Pyropia columbina* (Montagne) W.A.Nelson 2011 were collected on Cocholgüe beach, Concepcion (Chile, 36°35′37″ S; 75°58′43″ W) in February 2021 and *Gelidium corneum* (Hudson) J.V.Lamouroux and the marine lichen *Lichina pygmaea* (Lightfoot) C.Agardh were sampled in October 2020 on Las Palomas Island, Tarifa (Cadiz, Spain; 36°00′01.9″ N/05°36′33.2″). Samples were transported to the laboratory in a portable fridge at 4 °C, washed to remove salts and sediments, frozen at −80 °C, and freeze-dried (LyoQuest, Telstar, Bensalem, PA, USA).

### 3.3. Preparation of Algal Extracts

Lyophilized samples were ground and extracted in water (H_2_O). For extraction, 30 g of dry biomass (DB) was homogenized in 1 L of H_2_O using a blender (except in the case of *P. columbina*, where 60 g was extracted in 2 L of H_2_O). The mixtures were incubated in a water bath at 45 °C with constant agitation for 2 h. Then, the extracts were filtered and centrifuged. The biomass obtained after centrifugation was re-extracted three times. Finally, the extracts were concentrated in a rotary evaporator (R-210, Büchi, Switzerland) to remove part of the water, frozen at −80 °C, and lyophilized. The extraction yield was calculated using the following formula: Y = (W_DE_/W_DB_) ∗ 100, where W_DE_ is the weight of dry extract, and W_DB_ is the weight of dry biomass. The dry extracts (DE) were stored in the dark and with silica gel until their analysis.

### 3.4. MAAs Identification and Quantification

MAAs present in the extracts were identified by high-performance liquid chromatography (HPLC; Agilent 1100 Series, Santa Clara, CA, USA) according to Korbee-Peinado et al. [69] and Chaves-Peña et al. [55], with some modifications. For this purpose, 5 mg of DE was dissolved in 5 mL of distilled H_2_O. Separation was performed using a C18 column (Aqua^®^ 5 μm, 250 × 4.6 mm, Phenomenex, Aschaffenburg, Germany), with an isocratic flow (0.5 mL min^−1^) and a mobile phase of 2.5% methanol and 0.1% acetic acid in distilled H_2_O. Detection was performed using a photodiode array detector (DAD) 330 nm. Secondary standards were used to identify the MAAs, and quantification was based on the molar extinction coefficients (ε) of the different MAAs [70,71]: palythine (320) = 36,200 M^−1^ cm^−1^; asterina-330 (330) = 43,800 M^−1^ cm^−1^; shinorine (332) = 44,668 M^−1^ cm^−1^; porphyra-334 (334) = 42,300 M^−1^ cm^−1^; and mycosporine-serinol (310) = 25,516 M^−1^ cm^−1^. The following formulas were used for the quantification and are based on the Beer–Lambert Law:

MAAs (mg g^−1^) = (A × F)/(E × 60 × V_inj_ × DW)

MAAs (mg mL^−1^) = (A × F)/(E × 60 × 10^3^ × V_inj_)

where:

A is the area of the peak (mUA seg^−1^);

F is flux in the HPLC (mL min^−1^);

E is the extinction coefficient (L g^−1^ cm^−1^);

V_inj_ is the injection volume in the HPLC.

### 3.5. Isolation of MAAs

#### 3.5.1. Apparatus

For the isolation of the different MAAs, high-performance countercurrent chromatography (HPCCC Spectrum model, Dynamic Extractions Ltd., Slough UK) was used. The HPCCC was equipped with a 134 mL column (PTFE bore tubing = 3.2 mm). The column rotation was controlled by a speed regulator and the temperature of the column was controlled using a chiller (H50/H150, LabTech Srl., Sorisole Bergamo, Italy). The liquid phases were pumped through the column using a Q-Grad pump (LabAlliance, State College, PA, USA). The separation process was monitored using a UV-VIS spectrophotometer (Sapphire, ECOM spol. s.r.o., Prague, Czech Republic), working at a wavelength of 310 and 330 nm (depending on the MAAs) and recorded and analysed by EZChrom SI software (Agilent Technologies, Pleasanton, CA, USA).

#### 3.5.2. Selection of the Biphasic Solvent System

The biphasic solvent systems tested contained different combinations of ethanol (EtOH), acetonitrile (ACN), acetone (Acet.), saturated solution of (NH_4_)_2_SO_3_ in H_2_O (Sat(NH_4_)_2_SO_3_), saturated solution of NaCl in H_2_O (Sat(NaCl)), and H_2_O (Table 1). Ten mL of each of the different systems was prepared to analyse their physical and chemical properties. The closed tubes containing the biphasic solvent systems were mixed (3s vortexing) to measure the time required for the two phases to form two clear immiscible phases (settling time). The density difference was calculated by measuring the weight of 1 mL of each phase. To determine the partition coefficient (*K*) of each compound, 2 mg of the DE was mixed with 1 mL of the upper phase (UP) and 1 mL of the lower phase (LP). After mixing and leaving to equilibrate during 15 min, the two phases were analysed by HPLC (as explained in Section 3.3). The *K* value was calculated as the ratio of the peak areas (*K* = A_UP_/A_LP_, where A_UP_ is the area of the compound in the upper phase, and A_LP_ is the area of the same compound in the lower phase).

#### 3.5.3. Separation Procedure

The selected biphasic solvent system (EtOH:ACN:Sat(NH_4_)_2_SO_3_:H_2_O; 1:0.5:1:1 *v*:*v*:*v*:*v*) was prepared in a decanting funnel. The mixture was shaken vigorously, and once two clear phases appeared, they were separated and used as mobile (LP) and stationary phase (UP) in the reverse mode of the HPCCC. Before isolation, different parameters of HPCCC were optimized: the flow rate, the volume of the loop, and the sample loading per run. The resolution of the peaks (*R*) in the different experiments was mathematically calculated as *R* = 1.18 ∗ (Rt_2_ − Rt_1_)/(W_2_ + W_1_), where Rt is the retention time of each peak (measured in the middle of the peaks) and W is the weight of the peaks, measured at the middle height of the peaks. For the separation procedure, the HPCCC column was filled with the stationary phase at 10 mL min^−1^, then the column rotational speed was increased to 16,000 rpm, and the temperature was set to 30 °C. Then, the mobile phase was pumped at 3 mL min^−1^ until the hydrodynamic equilibrium was reached in the column. For a sample preparation, 1 g of dry *P. columbina* extract was dissolved in 6 mL LP, which corresponded to the loop volume. On the other hand, 200 mg of *G. corneum* extract was dissolved in 2 mL LP, while 1.2 g of *L. pygmaea* was dissolved in 6 mL LP. The a priori retention time of the target compounds during the HPCCC separation process was estimated as previously described [72]. In multiple injections, an elution–extrusion mode was used, in which all stationary phase was replaced [40]. The fractions obtained were manually collected and subsequently analysed by HPLC (as Section 3.3) for the recovery calculation.

### 3.6. Desalting

Size-exclusion chromatography was performed to remove most of the ammonium sulphate in the MAA-enriched fractions. Since ammonium sulphate precipitates with organic solvents, such as methanol, the MAA-enriched fractions were purified four times with pure methanol to remove as much ammonium sulphate as possible. Then, the samples were concentrated and 300 μL of the isolated MAAs were injected into the chromatographic column (52 cm length and 2 cm diameter) filled with Sephadex G-10 (Cytiva, Marlborough, MA, USA) at a flow rate of 0.5 mL min^−1^. Conductivity (LAQUAtwin EC-22, Horiba, Kyoto, Japan) and UV spectra in the range of 280–400 nm (UV-2700i, Shimadzu, Duisburg, Germany) were measured from each aliquot collected. Two fractions were separated from each injection into the column: one without or with a very small amount of ammonium sulphate (“desalted MAAs”) and another one with ammonium sulphate (“salted MAAs”). The fractions with ammonium sulphate (“Salted MAAs”) were dried under vacuum (Speed- Vac SPD210 Vacuum Concentrator, Thermo scientific, Waltham, MA, USA), redissolved in 300 μL, and reinjected into the column. This step was performed 4 times. The desalted samples were also analysed by HPLC (as Section 3.3) for recovery calculation.

### 3.7. Confirmation of the Chemical Identity of the Target Compounds

The chemical identity of the target compounds was confirmed by electrospray ionization mass spectrometry (HPLC-ESI-MS; Orbitrap Q-Exactive, Thermo Scientific S.L., Bremen, Germany), and by nuclear magnetic resonance (NMR; Avance III 500 MHz, Bruker, Fällanden, Switzerland). The HPLC-ESI-MS was performed at the premises of the Research Support Central Services (SCAI) of the University of Malaga. The spectral range was recorded between 70–700 *m*/*z* in positive and negative mode. The desolvation gas temperature was 230 °C, with a gas flow rate of 40 mLmin^−1^, auxiliary gas rate of 15 mL/min, and sweep gas rate of 2 m min^−1^. The spray voltage was set at 3.0 kV (positive) and 2.8 kV (negative). The compounds were identified by their exact mass.

For NMR analyses, the ^1^H spectra were recorded at 500 MHz and the ^13^C spectra at 125 MHz, and the compounds were dissolved in deuterated dimethyl sulfoxide (DMSO-d_6_), with residual solvent peaks at δ = 2.50 (DMSO) ppm for ^1^H and δ = 39.5 (DMSO) ppm for ^13^C.

## 4. Conclusions

In conclusion, five different MAAs were isolated by sequential multiple injections using HPCCC. As far as we know, this work is the first to report the separation of two of them—palythine and asterina-330. This study may open future perspectives for the development of new biological UV filters that protect in the UV-B and UV-A regions of the spectrum. The known photo- and thermostability of MAAs, and their multifunctional properties (UV protection, antioxidant, anti-inflammatory, DNA protection or anti-aging) make MAAs potential candidates for the new generation of biological UV filters that are healthier and more environmentally friendly, compared to physical or chemical filters. In addition, this method can serve as a reference for the development of optimized separation methods to obtain standards for qualitative and quantitative chemical analysis, since MAA standards are not yet available on the market.

## Figures and Tables

**Table 1:** Physical and chemical properties of the different biphasic solvent systems tested: phase volume ratio (UP:LP), settling time, density differences and partition coefficient values (*K*), for the isolation of mycosporine-like amino acids from three different extracts (two marine red macroalgae (*Pyropia columbina* and *Gelidium corneum*), and a marine lichen (*Lichina pygmaea*)).

Composition and Relative Proportion (*v*:*v*:*v*:*v*)		Ratio UP:LP	Settling Time (s)	Density Difference (g mL^−1^)	*P. columbina*		*G. corneum*		*L. pygmaea*
					*K* Values		*K* Values		*K* Values
					1	2	3	4	5
1	EtOH-ACN-Sat(NH_4_)_2_SO_4_-H_2_O (1:0.5:1:1)	1.80	22	0.3434	0.37	0.88	0.63	0.80	1.62
2	EtOH-ACN-Sat(NH_4_)_2_SO_4_-H_2_O (0.5:0.5:1:1)	0.82	20	0.2208	0.22	0.70	0.59	0.64	1.01
3	ACN-Sat(NH_4_)_2_SO_4_-H_2_O (0.5:1:1)	0.16	19	0.1985	-	-	-	-	-
4	EtOH-Sat(NH_4_)_2_SO_4_-H_2_O (1:1:1)	1.22	29	0.2359	0.89	1.31	0.74	0.87	1.39
5	EtOH-Acet-Sat(NH_4_)_2_SO_4_-H_2_O (1:0.5:1:1)	1.92	20	0.3492	0.22	0.99	0.79	0.79	1.71
6	EtOH-Acet-Sat(NH_4_)_2_SO_4_-H_2_O (0.5:0.5:1:1)	1.22	24	0.2636	0.24	0.97	0.75	0.55	1.56
7	Acet-Sat(NH_4_)_2_SO_4_-H_2_O (0.5:1:1)	0.59	30	0.1836	0.42	0.37	0.63	0.89	1.19

EtOH: ethanol; ACN: acetonitrile; Sat(NH_4_)_2_SO_4_: saturated solution of ammonium sulfate in water; Acet: acetone, UP: upper phase; LP: lower phase.

**Table 2:** Optimization of the flow rate (mL min^−1^) of the mobile phase against the stationary phase retention (%) in high-performance countercurrent chromatography (HPCCC) performance. Biphasic solvent system: EtOH-ACN-Sat(NH_4_)_2_SO_4_-H_2_O (1:0.5:1:1).

Flow Rate (mL min^−1^)	Stationary Phase Retention (%)	Pressure (psi)	Phase Lost due to Overpressure
1	79.1	110	
2	77.6	130	
3	77.6	145	
4	77.6	175	+
5	74.6	190	+
6	73	198	++
7	72.5	205	++
8	-	213	+++
9	-	220	+++
10	-	235	++++

+ means the amount of phase lost, a increasing number of + means an increasing phase lost.

**Table 3:** Parameters obtained from the different optimization procedures for high-performance countercurrent chromatography (HPCCC) performance for isolation of mycosporine-like amino acids. Stationary phase (*Sf*) lost in equilibrium and in run (%), pressure in equilibrium and run (psi), peak resolution (Rs), and loop capacity (mL) tested.

Experiment	Sample Loading (mg)	Sf Loss in Eq (%)	Sf Loss in Run (%)	Pressure in Eq (psi)	Pressure in Run (psi)	Peak Resolution (Rs)	Loop Capacity (mL)
*Pyropia columbina*							
A	200	28.4	11.9	138	117	1.80	3
B	400	28.4	17.0	145	122	1.52	3
C	600	38	28	145	115	1.36	3
D	800	40	32	145	108	1.25	6
E	**1000**	**40**	**32**	**145**	**110**	**1.18**	**6**
F	1200	40	34	145	118	0.99	6
*Gelidium corneum*							
G	100	36	4	140	125	1.36	2
H	**200**	**38**	**4**	**150**	**136**	**1.01**	**2**
I	300	38	8	152	138	0.96	2
*Lichina pygmaea*							
J	**1200**	**40**	**40**	**155**	**120**	**-**	**6**

## Data Availability

Not applicable.

## Supplementary Materials

The following supporting information can be downloaded at: https://www.mdpi.com/article/10.3390/md21060357/s1, Figure S1: Absorbance at 334 nm (maximum absorbance of porphyra-334) and conductivity measurement during the porphyra-334-enriched fraction desalting process using Sephadex G-10. In which, two different fractions were obtained, a desalted MAA-enriched fraction (red) and a salted MAA-enriched fraction (blue); Figure S2: Diagram with the amount of mycosporine-like amino acids (MAAs) and recovery rates (%) of the five isolated MAAs (shinorine, porphyra-334, palythine, asterina-330 and mycosporine-serinol) during all the steps of the isolation process (HPCCC procedure, cleanings with methanol and permeation in Sephadex G-10); Figure S3: Mass spectrum (70–700 *m*/*z*) of the different mycosporine-like amino-acid-enriched fractions obtained from *Pyropia columbina*, *Gelidium corneum* and *Lichina pygmaea* extracts. (A) Shinorine-enriched fraction, (B) porphyra-334-enriched fraction, (C) palythine-enriched fraction, (D) asterina-330-enriched fraction and (E) mycosporine-serinol-enriched fraction; Figure S4: ^1^H-NMR and ^13^C-NMR spectrum of the different mycosporine-like amino-acid-enriched fractions obtained from *Pyropia columbina*, *Gelidium corneum* and *Lichina pygmaea* extracts. (A) Shinorine-enriched fraction, (B) porphyra-334-enriched fraction, (C) palythine-enriched fraction, (D) mycosporine-serinol-enriched fraction.
