# Supplementary material for: Isolation of Mycosporine-like Amino Acids from Red Macroalgae and a Marine Lichen by High-Performance Countercurrent Chromatography: A Strategy to Obtain Biological UV-Filters
Source: Mar Drugs. 2023 Jun 10;21(6):357. doi: 10.3390/md21060357 (PMC10303195; doi:10.3390/md21060357)
Supplement: Supplementary file 1 [file marinedrugs-21-00357-s001.zip › marinedrugs-2372287-supplementary.pdf]

Supplementary material

Isolation of mycosporine-like amino acids from red macroalgae and a marine lichen by high performance countercurrent chromatography: a strategy to obtain biological UV-filters

Julia Vega[1*], Daniela Bárcenas-Pérez[2,3], David Fuentes-Ríos[4], J. Manuel López-Romero[4], Pavel Hrouzek[2], Felix L. Figueroa[1] and José Cheel[2*]

[1] Universidad de Málaga, Instituto Andaluz de Biotecnología y Desarrollo Azul (IBYDA), Centro Experimental Grice Hutchinson, Lomas de San Julián, 2, 29004-Málaga, Spain
[2] Laboratory of Algal Biotechnology – Centre ALGATECH, Institute of Microbiology of the Czech Academy of Sciences, Opatovický mlýn, 379 81 Třeboň, Czech Republic
[3] Faculty of Science, University of South Bohemia, Branišovská 1760, 370 05 České Budějovice, Czech Republic
[4] Department of Organic Chemistry, Faculty of Sciences, University of Malaga, Campus de Teatinos s/n, 29071 Malaga, Spain
* corresponding author: juliavega@uma.es; jcheel@alga.cz

Figure S1. Absorbance at 334nm (maximum absorbance of porphyra-334) and conductivity measurement during the porphyra-334 enriched fraction desalting process using Sephadex G-10. In which, two different fractions were obtained, a desalted MAAs enriched fraction (red) and a salted MAAs enriched fraction (blue).

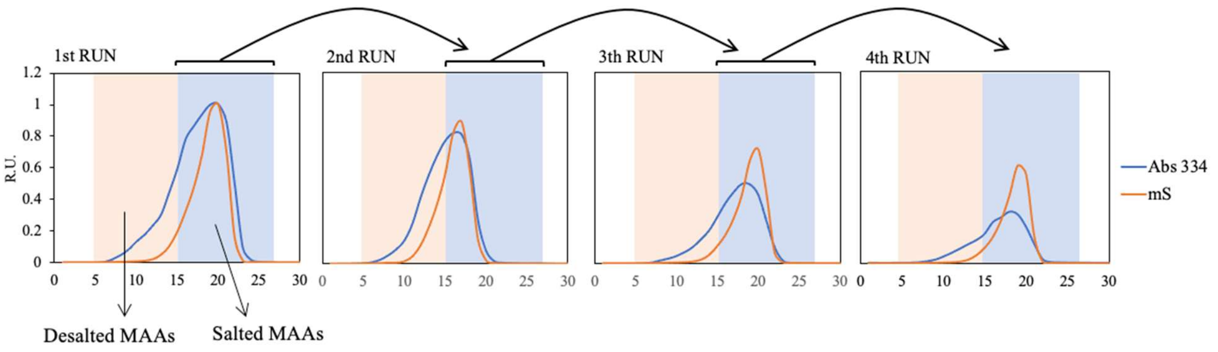

Figure S2. Diagram inlcuding the amount and recovery rates (%) of the five isolated mycosporine-like amino acids (MAAs: shinorine, porphyra-334, palythine, asterina-330 and mycosporine-serinol) from two red macroalgae (*Pyropia columbina* and *Gelidium corneum*) and a marine lichen (*Lichina pygmaea*) during all the steps of the isolation process (HPCCC procedure, cleanings with methanol and permeation in Sephadex G-10).

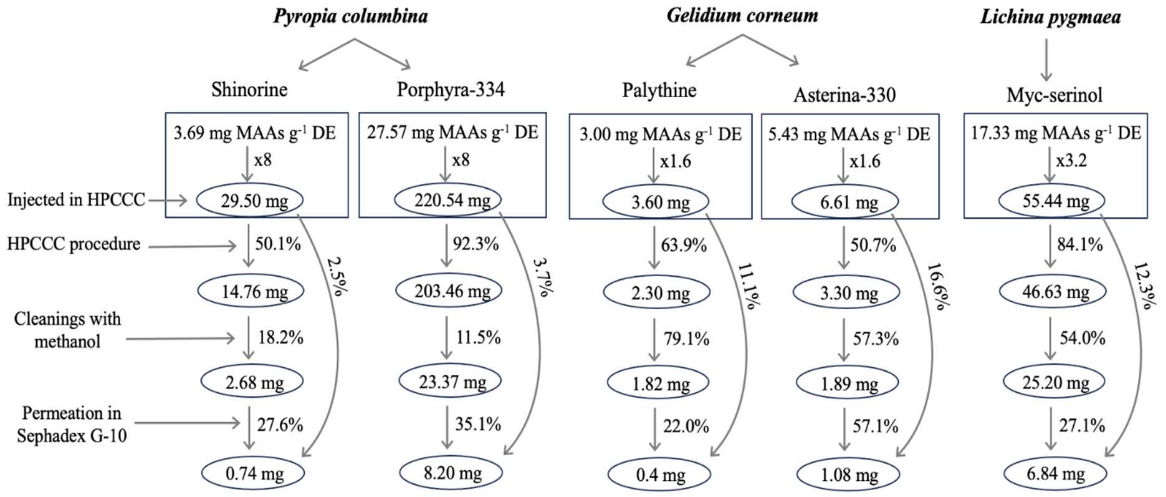

Figure S3. Mass spectrum (70-700 m/z) of the different mycosporine-like amino acids enriched fractions obtained from *Pyropia columbina*, *Gelidium corneum* and *Lichina pygmaea* extracts. A) Shinorine enriched fraction, B) Porphyra-334 enriched fraction, C) Palythine enriched fraction, D) Asterina-330 enriched fraction and E) Mycosporine-serinol enriched fraction.

A) SH-enriched fraction

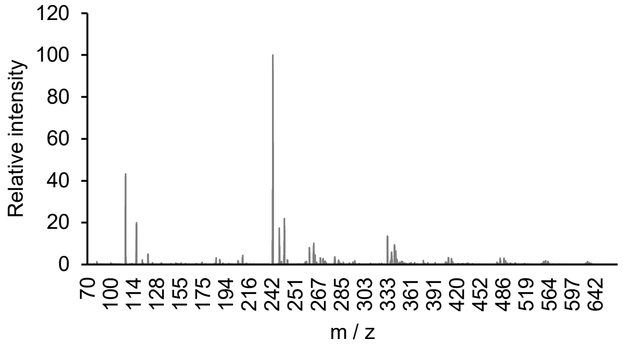

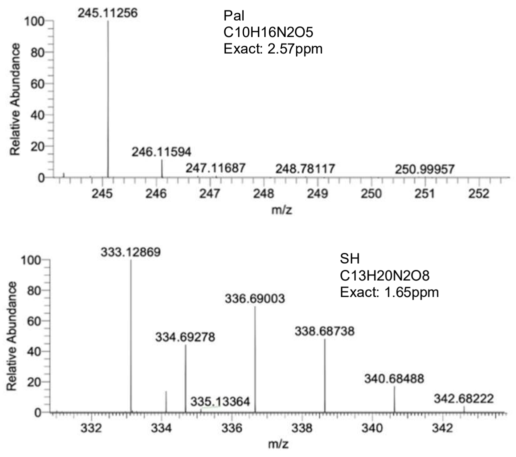

B) P-334-enriched fraction

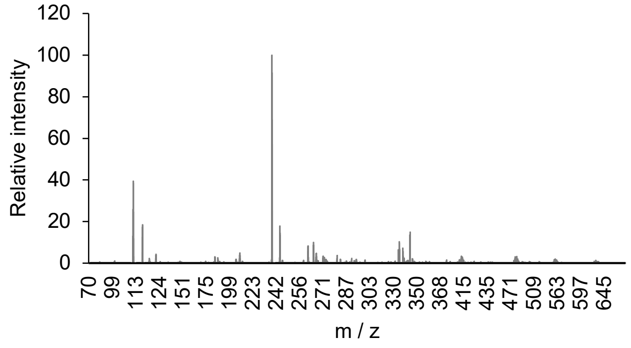

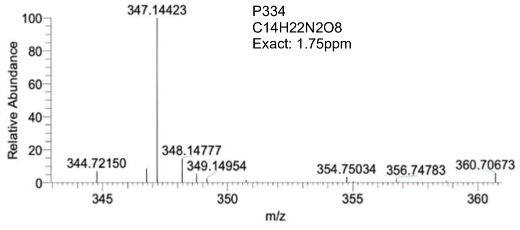

C) Pal-enriched fraction

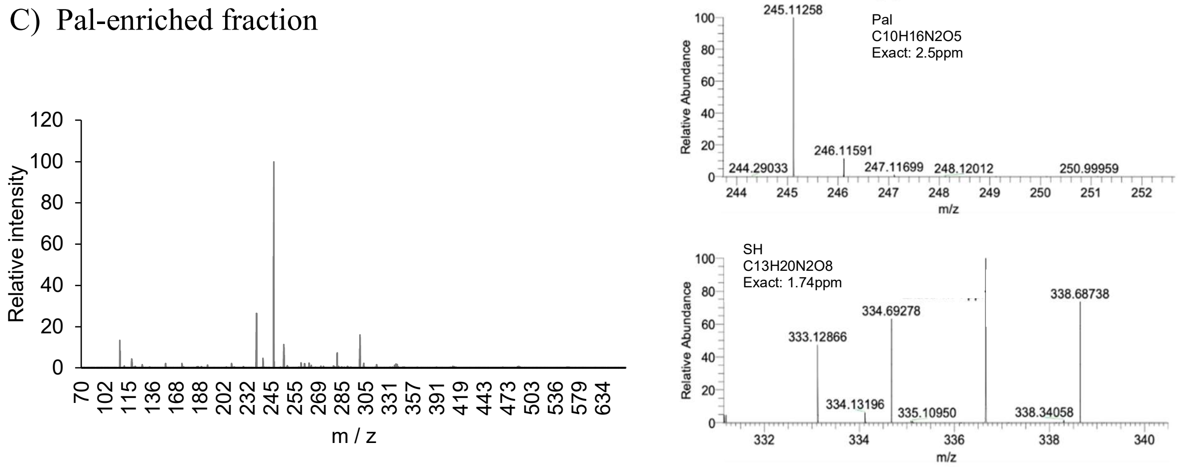

D) Ast-330-enriched fraction

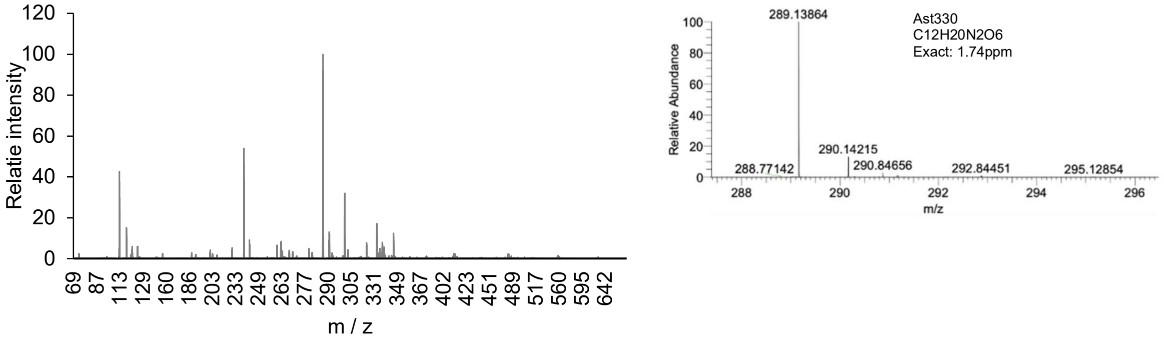

E) M-ser-enriched fraction

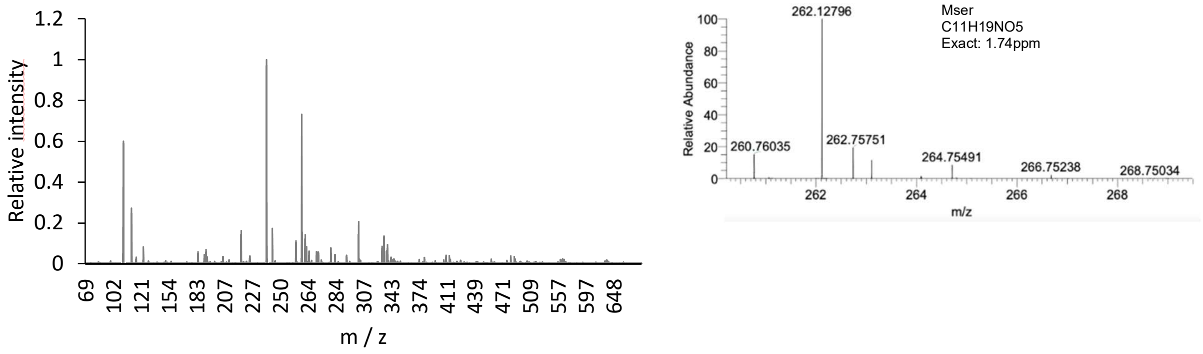

Figure S4. ^{1}H-NMR and ^{13}C-NMR spectrum of the different mycosporine-like amino acids enriched fractions obtained from *Pyropia columbina*, *Gelidium corneum* and *Lichina pygmaea* extracts. A) Shinorine enriched fraction, B) Porphyra-334 enriched fraction, C) Palythine enriched fraction, D) Mycosporine-serinol enriched fraction.

A

**A / ^{1}H NMR**			
**Chemical Shift** (ppm)	**Multiplicity**	**Hydrogens**	**Integral**
2.3-2.4	m	2 x CH_2	4
2.4	m	CH_2N	2
3.06	s	CH_2O	2
3.11	s	CH_2O	2
3.70	s	OCH_3	3
4.3-4.6	m	CH-CH_2O	2

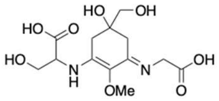

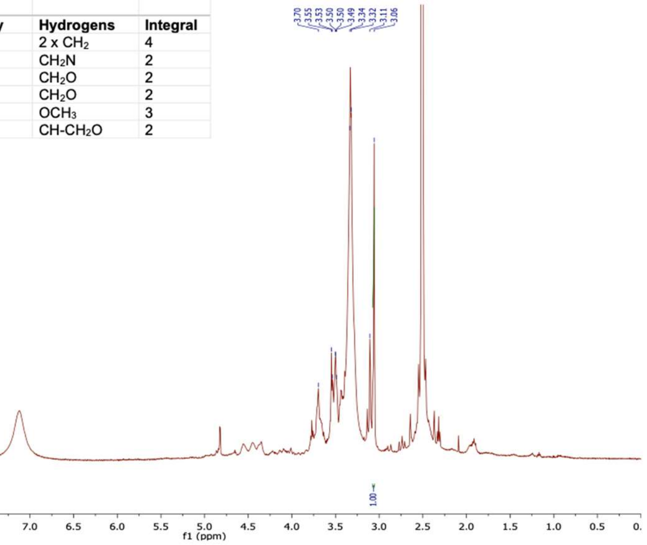

^{1}H-NMR of SH enriched fraction (DMSO-d_6)

**A / ^{13}C NMR**	
**Chemical Shift** (ppm)	**Carbon**
52.7	CH_2
55.8	CH_2
61.0	CH
62.8	CH
62.9	CH
70.2	CH
71.8	CH
81.1	Cuat
124.9	C=C
157.8	C=C
165.0	C=N
177.1	C=O
179.0	C=O

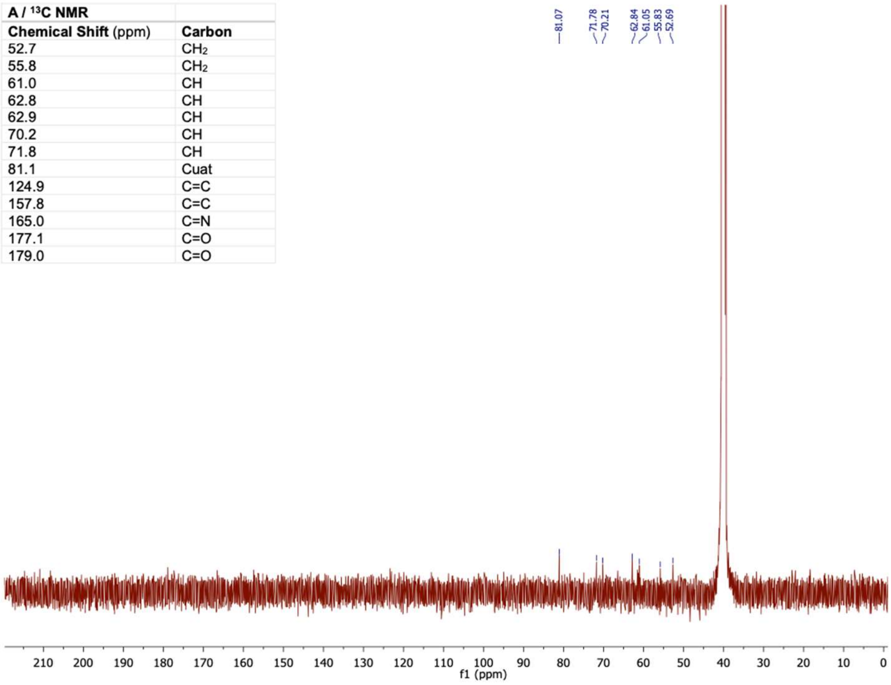

^{13}C-NMR of SH enriched fraction (DMSO-d_6)

B

B / ¹H NMR			
**Chemical Shift** (ppm)	**Multiplicity**	**Hydrogens**	**Integral**
1.05	d, 2.5 Hz	CH_3	3
2.5-2.6	m	2 x CH_2	4
2.7	m	CH_2N	2
3.2-3.3	m	2 x CH_2O	4
3.56	s	OCH_3	3
4.0	m	NCH-CHO	2

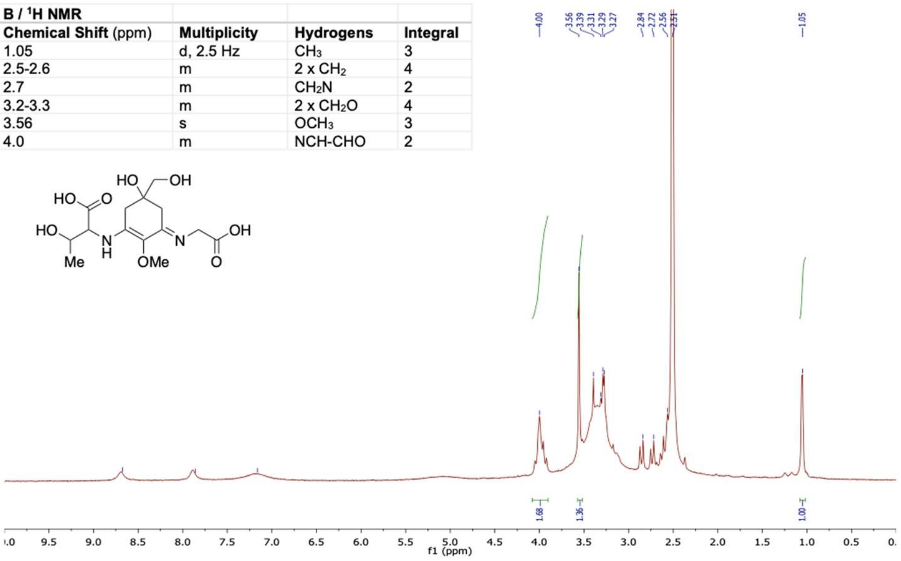

¹H-NMR of P-344 enriched fraction (DMSO-d_6)

B / ¹³C NMR	
**Chemical Shift** (ppm)	**Carbon**
19.9	CH
33.2	CH_2
33.8	CH_2
58.9	CH
61.1	CH
62.8	CH
66.7	CH
67.9	CH
70.8	Cuat
125.0	C=C
147.8	C=C
159.9	C=N
176.5	C=O
180.1	C=O

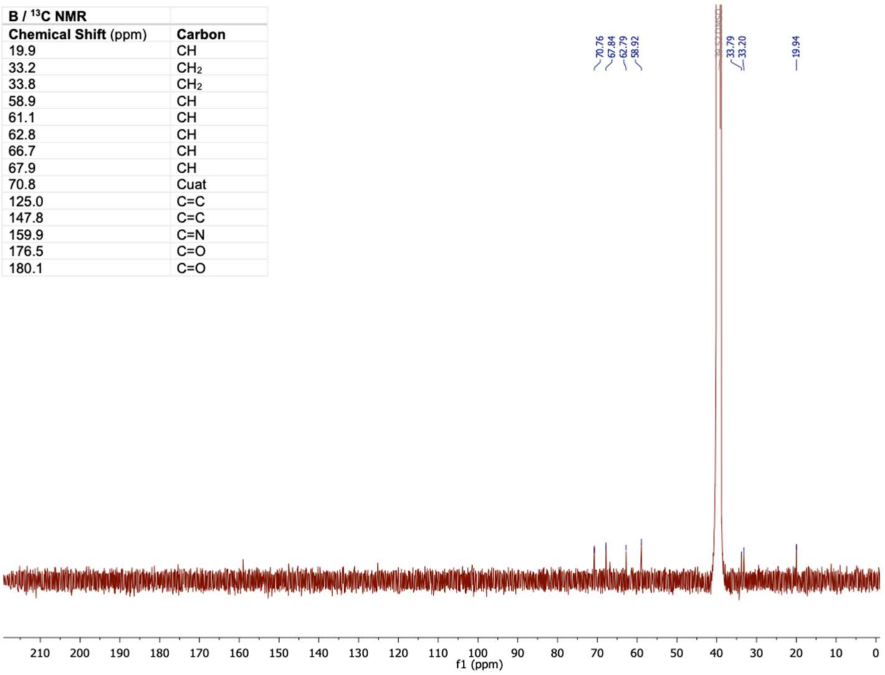

¹³C-NMR of P-344 enriched fraction (DMSO-d_6)

C

C / ^{1}H NMR			
**Chemical Shift** (ppm)	**Multiplicity**	**Hydrogens**	**Integral**
2.6-2.8	m	2 x CH_2	4
3.3	s	CH_2N	2
3.6	s	OCH_3	3

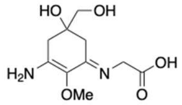

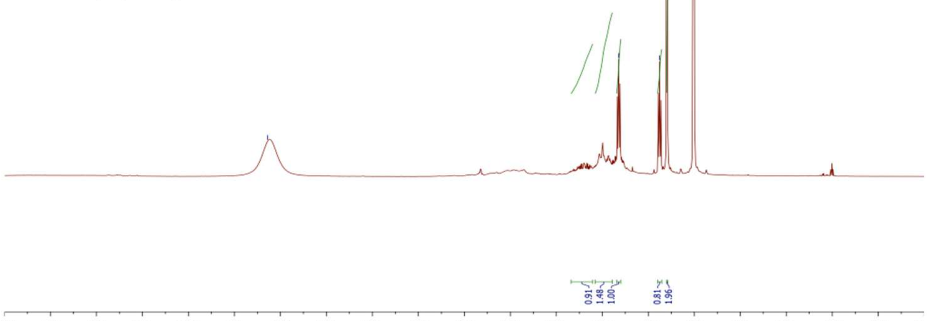

^{1}H-NMR of Pal enriched fraction (DMSO-d_6)

C / ^{13}C NMR	
**Chemical Shift** (ppm)	**Carbon**
61.0	CH_2
63.5	CH_2
68.2	CH
70.2	CH
70.8	CH
81.0	Cuat
100.0	C=C
105.0	C=C
159.9	C=N

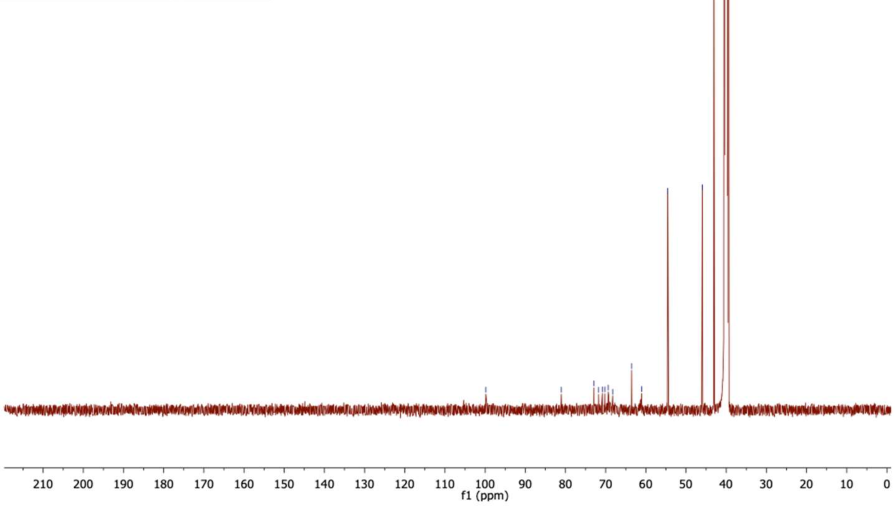

^{13}C-NMR of Pal enriched fraction (DMSO-d_6)

D

D / ¹H NMR			
**Chemical Shift** (ppm)	**Multiplicity**	**Hydrogens**	**Integral**
2.10-2.13	m	2 x CH_2	4
3.2	m	CHN	1
3.4-4.1	m	3 x CH_2O	6
3.59	s	OCH_3	3

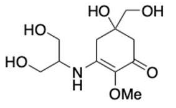

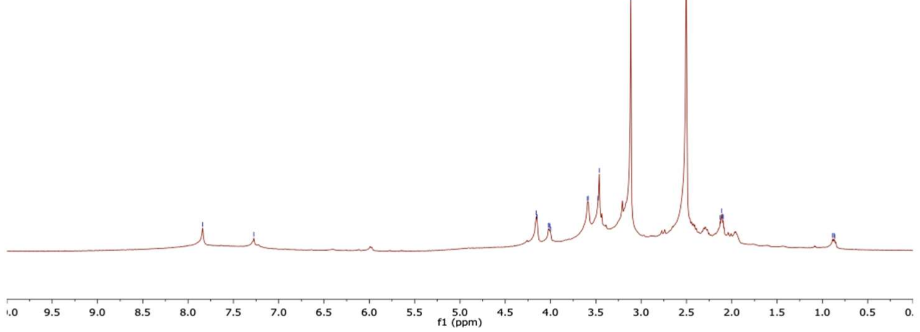

^{1}H-NMR of M-ser enriched fraction (DMSO-d_6)

D / ¹³C NMR	
**Chemical Shift** (ppm)	**Carbon**
25.2	CH_2
29.6	CH_2
53.5	CH
55.5	CH
61.4	CH
72.1	Cuat
127.8	C=C
130.4	C=C
175.1	C=O

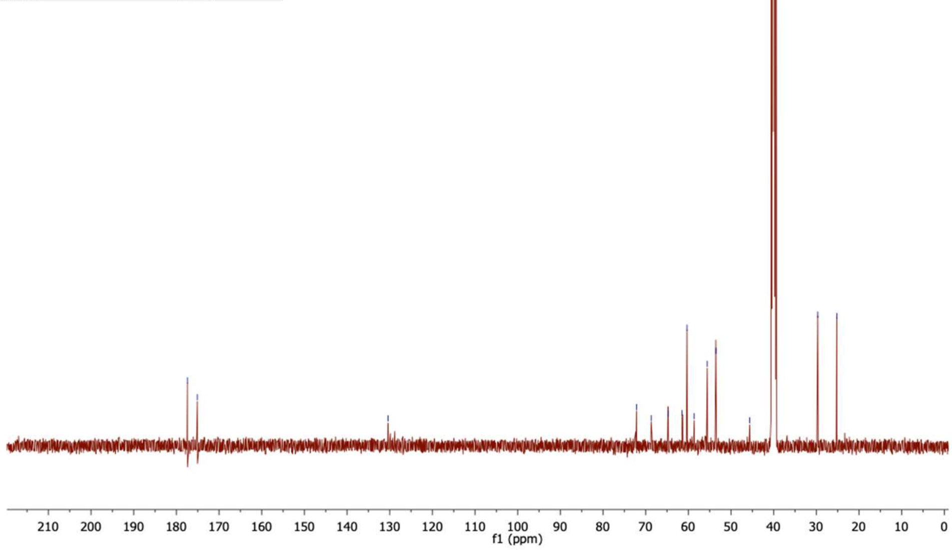

^{13}C-NMR of M-ser enriched fraction (DMSO-d_6)
